# Validity and Reliability of an Inertial Sensor-Based Knee Proprioception Test in Younger vs. Older Adults

**DOI:** 10.3389/fspor.2019.00027

**Published:** 2019-09-18

**Authors:** Anna Lina Rahlf, Evi Petersen, Dominique Rehwinkel, Astrid Zech, Daniel Hamacher

**Affiliations:** ^1^Department of Exercise Physiology, Institute of Sport Science, Friedrich Schiller University of Jena, Jena, Germany; ^2^Department of Sports, Physical Education and Outdoor Studies, University of South-Eastern Norway, Kongsberg, Norway

**Keywords:** proprioception, inertial sensor, joint position sense, test-retest reliability, older adults

## Abstract

Diminishing proprioception caused by aging effects is associated with a higher risk to fall. However, existing measurement systems of proprioception are often expensive, time-consuming, or insufficient regarding reliability evaluation. Inertial sensor-based systems could address these issues. Consequently, this study sought to develop and evaluate an inertial sensor-based joint position sense test. Thereto, intra-session and inter-day test-retest reliability were investigated in a cross-over design. Twenty healthy younger (age: 22 ± 3 years) and 20 healthy older adults (age: 65 ± 5 years) participated in the study. We calculated the mean of the absolute error, the signed error, and the standard deviation of the signed error. Test-retest reliability was quantified by using the intraclass correlation coefficient as well as the bias and limits of agreement. To evaluate the possibility of capturing aging effects, and correspondingly a validation of the system, we calculated Cohen's *d*. For the intra-session reliability, fair to good agreements were achieved for the absolute and relative error in all target ranges. Compared to younger adults, we registered a declined joint position sense in older adults with high effects observed for the absolute error in a target range of 15–25 and 35–45° as well as for the variable error in the target ranges of 35–45 and 55–65°. We suggest that inertial sensor-based joint position sense tests are reliable and capable to measure aging effects on proprioception, and are therefore a low-cost and mobile alternative to existing methods.

## Introduction

Up to 50% of older adults fall at least once per year (Tinetti et al., 1988; Hausdorff et al., 2001; Inouye et al., 2009). One risk factor that can cause falls is an impaired balance (Tinetti et al., 1988), which again depends on a well-functioning proprioceptive perception. Proprioception is known to be essential for joint stabilization and provides the basis for an adequate sensorimotor control (Riemann and Lephart, 2002; Laube, 2009; Proske and Gandevia, 2012). For instance, small variations in joint angles are mainly perceived by proprioceptors (Fitzpatrick and McCloskey, 1994). With an increase in age such proprioceptive performance capability decreases (Wingert et al., 2014). Thus, in older adults, diminishing proprioception is widely discussed to contribute to the increased risk of falling (Proske and Gandevia, 2012; Suetterlin and Sayer, 2013). Furthermore, proprioception of the joint is used to assess the risk of falling (Lord et al., 2003). To summarize, the age-related decline in proprioception moderates a couple of geriatric syndromes e.g., falls (Suetterlin and Sayer, 2013). Probably, due to the lack of reliable, feasible, and mobile methods to assess proprioception (Benjaminse et al., 2009), such methods are still seldom used in clinical practice (Suetterlin and Sayer, 2013). A reliable assessment of proprioception performance status is therefore beneficial within the scope of evaluating fall risk (Hurley et al., 1998).

In general, three submodalities have been described to assess proprioception: (a) joint position sense, (b) kinesthesia (e.g., the threshold to detection of passive motion) or (c) sense of tension/force (Riemann et al., 2002; Suetterlin and Sayer, 2013). Besides most measure approaches require complex and expensive technical equipment (Suetterlin and Sayer, 2013), established methods lack to differentiate between healthy subjects and patients with specific diseases (Riemann et al., 2002) or participants of different age, e.g., younger vs. older adults (Pickard et al., 2003). Reasons for this can be traced back to insufficient reliability of these methods (Benjaminse et al., 2009) as well as the general existence of various confounders (Riemann et al., 2002).

Acceptable reliability of a joint position sense test has been reported in the study of Arvin et al. (2015). The authors used a camera-based system to capture the reproducibility of the knee angle. More precisely, subjects actively moved their knees into a targeted joint angle, first to memorize the reference position and second to replicate this position in a blindfolded condition. However, camera-based systems come with disadvantages regarding practicability as they are often expensive, require well-equipped labs, and test setups can be relatively time-consuming. Thus, for many assessment situations, for instance, in clinical contexts, this method is inefficient.

Inertial sensors allow for an inexpensive measurement of biomechanical parameters, which has been successfully applied to the analysis of gait and postural balance (Hamacher et al., 2014; King et al., 2014). Compared to what is usually deemed the gold standard (e.g., optical, sonic, or magnetic capture systems), body-fixed-sensor based technologies (e.g., inertial sensors) do not require a permanent laboratory nor specialized testers with a sound technical background to reliably assess human movements (Zijlstra and Aminian, 2007). While the application of inertial sensors could improve the feasibility of evaluating proprioception, to the best of our knowledge, no inertial sensor-based joint position sense test for the knee joint does exist, yet.

Consequently, the aim of our study was to evaluate the developed system regarding (1) the test-retest reliability (stability) of different joint position sense measures over time. Since proprioception is known to decline with age (Relph and Herrington, 2016; Alahmari et al., 2017), we further (2) rated the criterion-related validity of different joint position sense measure by rating the ability to assess aging-effects. The reliability and validity of joint position sense measures might depend on the target angels chosen. However, there is little evidence on which target angle to use when evaluating joint position sense. Therefore, we also aimed at (3) rating reliability and validity for various target angle ranges. Until now, no optimal target angle to evaluate proprioception has been defined. The current target angles in the literature regarding joint position sense at the knee vary between 10 and 100° (Bullock-Saxton et al., 2001; Arvin et al., 2015; Irving et al., 2016; Relph and Herrington, 2016). For example, in the Physiological Profile Assessment (PPA) to assess fall risk of Lord et al. (2003), midrange angles of the knee were chosen. Furthermore, our preliminary tests showed that smaller angles (e.g., 10°) are practically challenging to test as the range for errors is small. We further observed that larger angles (e.g., 80°) lead to fatiguing effects. Therefore, we choose to determine the optimal target angles (15–25, 35–45, 55–65°) for assessing aging effects.

## Materials and Methods

### Subjects

We evaluated the inertial sensor-based joint position sense test by conducting an intra-session and an inter-day test-retest study. For this purpose, 20 female younger adults (age: 22 ± 3 years, height: 1.69 ± 0.07 m, weight: 64 ± 8 kg) and 20 female older adults (age: 65 ± 5 years, height: 1.63 ± 0.09 m, weight: 69 ± 7 kg) were included in the study. Exclusion criteria were any acute self-reported motor-functional impairments. For the older adults, an age of at least 60 years was an additional inclusion criterion. To test the test-retest reliability (stability) of joint position sense measures over time, all participants were tested twice during the first day (intra-session test-retest interval: 15 min) and once seven days later (inter-day test-retest interval: 7 days).

To rate the validity of the different measures, we compared both groups. All participants provided their written informed consent after they were informed about the research protocol, which itself complied with the principles of the Declaration of Helsinki and was approved by the local ethical committee (No. FSV 16/10).

### Measurement Procedure

**Figure 1:**
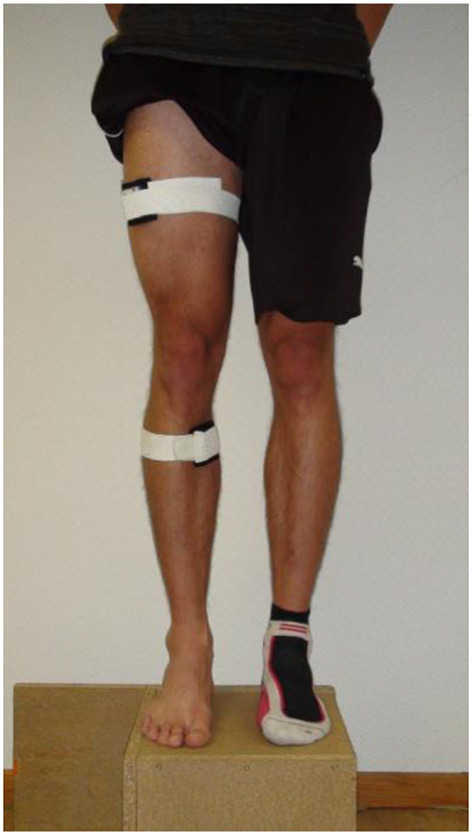
Sensor application.

The joint position sense was evaluated by using the reproducibility of the knee joint. To measure the knee angle, we used inertial sensors (MTw2, Xsens Technologies B.V., Enschede, The Netherlands) with a sampling rate of 100 Hz. Using elastic straps, the inertial sensors were attached medial and distal to the tibia tuberositas at the shank as well as to the right iliotibial tract at the middle of the thigh (Figure 1). The Xsens software development kit was used to access the orientation data in MATLAB in real-time (version 2014a, The MathWorks, Natick, USA). We placed a reference sensor on the ground and aligned the reference sensor local coordinate systems to the participant's sagittal, horizontal, and frontal plane. To calibrate, the participants were asked to stand in a normal upright position with legs hip-width apart. By using this calibration pose for both body-attached sensors, the sagittal plane was identified and knee flexion/extension in the sagittal plane could be measured in real-time.

**Figure 2:**
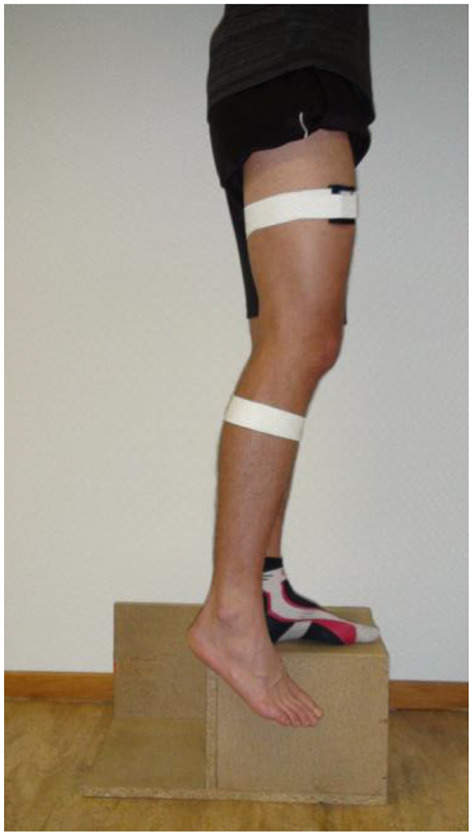
Starting position for active angle reproduction.

We adopted the measurement procedure reported by Arvin et al. (2015) and used an “active-active” reproduction technique. The participants stood on a wooden platform facing a wall with a distance of ~0.2 m. Keeping their eyes closed, participants stabilized their balance by placing their hands onto the wall. They were advised to stand on their left leg while their right leg pended freely aside from the wooden platform (Figure 2). The participants were then asked to slowly flex their right knee until the targeted angle was reached and the examiner said “stop”. After the leg completely stopped moving, the participants were instructed to memorize this knee angle for about 4 s. Once the participants verbally informed the examiner that they had memorized the reference knee angle, the exact knee angle was recorded. After the participants returned to the starting position, they were asked to replicate the memorized knee angle on their own. Once they assumed to have reached the memorized reference position, they informed the examiner again by saying “stop.” The presented angle was recorded.

In total, we tested three target angles within the ranges of (a) 15–25°, (b) 35–45°, and (c) 55–65° of knee flexion. In a previous study, Selfe et al. found no difference in active angle reproduction between 20 and 60° in patients (Selfe et al., 2006). Since the measured error depends on the degree of joint flexion (Edwards et al., 2016), and the fact that no target angle to analyze falls risk exists, we decided to investigate three target ranges. The sequence of target range was the same for each participant. The set of target ranges (a–c) was recorded 10 times resulting in 10 trials for each target range.

### Data Analysis and Statistics

For data analysis, we excluded the first two trials of each range as those were considered learning trials. For the remaining eight trials, we calculated (1) the mean of the absolute error of each trial (absolute error addressing only the absolute value of error), (2) the mean of the signed error (relative error calculating the mean of both positive and negative errors) and (3) the standard deviation of the signed error (variable error). Test-retest reliability was quantified by using the intraclass correlation coefficient (ICC 2.1) as well as the bias and limits of agreement (LoA) (Bland and Altman, 1986). Regarding the ICC, values of 0.0–0.40 were considered poor, 0.40–0.59 fair, 0.60–0.74 good, and 0.75–1.00 to be excellent (Cicchetti, 1994). To rate the validity, we used paired *t*-tests and calculated Cohen's *d* (d) to assess aging effects of younger vs. older adults.

## Results

The intra-day session reliability ranged from poor (ICC = 0.25) to good (ICC = 0.63). In detail, good values were achieved for the absolute (ICC = 0.63, *p* < 0.001) and relative error (ICC = 0.61, *p* < 0.001) within a target range of 15–25° as well as the absolute error (ICC = 0.63, *p* < 0.001) within a target range of 35–45°. The inter-day session reliability was lower and ranged from poor (ICC = 0.02) to fair (ICC = 0.52): fair values were achieved in the absolute (ICC = 0.50, *p* = 0.001) and relative error (ICC = 0.52, *p* < 0.001) within a target range of 15–25° as well as the relative error within a target range of 55–65° (Table 1).

**Table 1:** Test-retest reliability.

**Parameter**	**Intra-session**				**Inter-day**			
	**ICC**	***p***	**Bias**	**LoA**	**ICC**	***p***	**Bias**	**LoA**
**Target range: 15–25**° **of knee joint flexion**								
Absolute error	0.63 (good)	<0.001	0.57	3.61	0.50 (fair)	0.001	0.85	4.28
Relative error	0.61 (good)	<0.001	−0.70	4.07	0.52 (fair)	<0.001	−1.13	4.73
Variable error	0.25 (poor)	0.059	0.04	2.44	0.02 (poor)	0.549	0.10	2.69
**Target range: 35–45**° **of knee joint flexion**								
Absolute error	0.63 (good)	<0.000	−0.04	2.33	0.02 (poor)	0.538	0.24	3.35
Relative error	0.53 (fair)	<0.000	0.30	3.72	0.05 (poor)	0.387	0.04	4.65
Variable error	0.40 (fair)	0.006	0.00	2.32	0.18 (poor)	0.141	0.27	2.46
**Target range: 55–65**° **of knee joint flexion**								
Absolute error	0.42 (fair)	0.004	0.09	2.18	0.23 (poor)	0.083	0.25	2.30
Relative error	0.54 (fair)	<0.000	0.07	3.66	0.45 (fair)	0.002	0.37	3.73
Variable error	0.25 (poor)	0.062	−0.15	2.06	0.02 (poor)	0.548	0.06	2.31

Table 2 shows the results of the comparisons of younger vs. older adults. Large significant effects have been depicted for the absolute error (*d* = 0.96) within the target range of 15–25°, the absolute error (*d* = 0.89) and the variable error (*d* = 1.17) within the target range of 35–45° as well as the variable error (*d* = 0.89) within the target range of 55–65°.

**Table 2:** Comparison of different joint position sense measures in younger vs. older adults within the target ranges of 15–25, 35–45, and 55–65° of knee joint flexion.

**Parameter**	**Older adults**	**Younger adults**	**Older vs. younger adults**	
	**Mean (SD)**	**Mean (SD)**	***p***	**Cohens *d***
**Target range: 15–25**° **of knee joint flexion**				
Absolute error	4.36 (2.63)	2.47 (0.90)	0.007	0.96
Relative error	−3.69 (3.05)	−1.92 (1.35)	0.029	0.75
Variable error	2.64 (1.09)	2.05 (0.55)	0.043	0.69
**Target range: 35–45**° **of knee joint flexion**				
Absolute error	2.81 (1.09)	2.03 (0.59)	0.011	0.89
Relative error	−1.84 (1.38)	−1.08 (1.34)	0.098	0.55
Variable error	2.64 (0.88)	1.78 (0.55)	0.001	1.17
**Target range: 55–65**° **of knee joint flexion**				
Absolute error	2.30 (0.85)	1.88 (0.70)	0.105	0.54
Relative error	−0.84 (1.42)	−0.12 (1.61)	0.149	0.48
Variable error	2.37 (0.79)	1.75 (0.59)	0.010	0.89

## Discussion

To the best of our knowledge, we developed and evaluated the first inertial sensor-based joint position sense test using a test-retest design. Furthermore, the validity of different measures was assessed by comparing the joint position sense of healthy younger vs. older adults. Except for the variable error in the target range of 15–25 and 55–65°, the intra-session reliability reached at least fair to good agreements for the absolute and relative error in all target ranges.

To compare our results with the existing body of literature, we focus on active-active approaches to evaluate joint position sense. In these studies, fair to good intra-session reliability was reported for the absolute knee angle (Arvin et al., 2015; Clark et al., 2016), which is comparable to our results. While the intra-session reliability for the hip joint was inferior (ICC = 0.159–0.319) to our results, they reached poor to good inter-session reliability (ICC = −0.079–0.753) compared to their intra-session (Benjaminse et al., 2009) and our inter-session reliability. Besides camera-based measurement systems, various principles of measurements were used to quantify the joint position sense. For example, for the electromagnetic measure at the shoulder joint, a fair ICC of 0.4 was reported (Lönn et al., 2000). For the ankle joint position sense, excellent intra-session, as well as excellent inter-session reliability (ICC2,k: 0.83–1.00), were reported (Deshpande et al., 2003; You, 2005). To resume the discussion, we reached a comparable level of reliability. As an exception, the test-retest reliability was excellent for the ankle joint in other studies. However, these studies examined a different joint with a different measurement principle such as electromagnetic tracking systems or potentiometers.

In the comparing of younger vs. older adults, high effects were observed for the absolute error in a target range of 15–25 and 35–45° as well as for the variable error in the target ranges of 35–45 and 55–65°. The study of Selfe et al. (2006) found no difference in active angle reproduction between 20 and 60° in patients with patella femoral pain syndrome.

To validate the different measures, we also compared the joint position sense of younger vs. older adults. Since age effects are well-documented in a review (Goble, 2010), this is reasonable. Except for the relative error within the target range of 55–65°, we observed medium to large effects (*d* = 0.54–1.17) in all measures within all target ranges. Thus, the inertial sensor-based system is capable of detecting age-related effects. According to the effect size, the absolute error within the target ranges of 15–25 and 35–45° as well as the variable error within the target ranges of 35–45 and 55–65° are recommended for studies focusing age-related effects on the knees joint position sense.

In terms of limitations to our study, it should be mentioned that the generalizability of our findings is limited to healthy older adults and the measure of knee joints. Moreover, we only registered the active-active joint-position sense. Both calibration and testing were conducted during single-leg stance. Therefore, this setup might not be suitable for some older adults or patients. However, the setup can also be adjusted to perform the test in a sitting position. The use of an inertial sensor-based system that minimizes costs and time required for the measurements designates this system as relevant for fast screenings of larger cohorts. Furthermore, there are no specific requirements needed for laboratories. Thus, such tests can be conducted in various settings.

## Conclusion

Based on our results, we could show that inertial sensor-based joint position sense tests are reliable and capable to measure aging effects on proprioception. To rate the joint position sense of the knee joint within a cohort, the reliability was good for the absolute error in the target ranges of 15–25 and 35–45°' knee flexion as well as for the relative error in a target range of 15–25°. Furthermore, the variable error (target range: 35–45°' knee flexion) revealed the highest effect sizes (*d* = 1.17) and is consequently recommended for the comparison of distinct groups (e.g., younger vs. older adults). Besides focusing on improving decreased proprioception in impaired, elderly, or injured populations, further studies should assess the minimal clinical important difference to facilitate implementation into the clinical practice.

## Data Availability

The dataset for this manuscript is not publicly available, however, requests to access the datasets should be directed to Evi Petersen (evi.petersen@usn.no).

## Ethics Statement

All participants provided their written informed consent after they were informed about the research protocol, which itself complied with the principles of the Declaration of Helsinki and was approved by the local ethical committee Friedrich Schiller University Jena (No. FSV 16/10).

## Author Contributions

AR, EP, DR, AZ, and DH were fully involved in the study and preparation of the manuscript. Each of the authors has read and concurs with the content in the final manuscript.

### Conflict of Interest Statement

The authors declare that the research was conducted in the absence of any commercial or financial relationships that could be construed as a potential conflict of interest.
